# Efficient Remediation of *p*-chloroaniline Contaminated Soil by Activated Persulfate Using Ball Milling Nanosized Zero Valent Iron/Biochar Composite: Performance and Mechanisms

**DOI:** 10.3390/nano13091517

**Published:** 2023-04-29

**Authors:** Zihan Guo, Dong Wang, Zichen Yan, Linbo Qian, Lei Yang, Jingchun Yan, Mengfang Chen

**Affiliations:** 1 Key Laboratory of Soil Environment and Pollution Remediation, Institute of Soil Science, Chinese Academy of Sciences, Nanjing 210008, China; 2 University of Chinese Academy of Sciences, Beijing 100049, China; 3 Jiangsu Environmental Engineering Technology Co., Ltd., Nanjing 210019, China

**Keywords:** *p*-chloroaniline, zero valent iron/biochar, ball milling, persulfate, soil remediation

## Abstract

In this study, efficient remediation of *p*-chloroaniline (PCA)-contaminated soil by activated persulfate (PS) using nanosized zero-valent iron/biochar (B-nZVI/BC) through the ball milling method was conducted. Under the conditions of 4.8 g kg^−1^ B-nZVI/BC and 42.0 mmol L^−1^ PS with pH 7.49, the concentration of PCA in soil was dramatically decreased from 3.64 mg kg^−1^ to 1.33 mg kg^−1^, which was much lower than the remediation target value of 1.96 mg kg^−1^. Further increasing B-nZVI/BC dosage and PS concentration to 14.4 g kg^−1^ and 126.0 mmol L^−1^, the concentration of PCA was as low as 0.15 mg kg^−1^, corresponding to a degradation efficiency of 95.9%. Electron paramagnetic resonance (EPR) signals indicated SO_4_•^−^, •OH, and O_2_•^−^ radicals were generated and accounted for PCA degradation with the effect of low-valence iron and through the electron transfer process of the *sp*^2^ hybridized carbon structure of biochar. 1-chlorobutane and glycine were formed and subsequently decomposed into butanol, butyric acid, ethylene glycol, and glycolic acid, and the degradation pathway of PCA in the B-nZVI/BC-PS system was proposed accordingly. The findings provide a significant implication for cost-effective and environmentally friendly remediation of PCA-contaminated soil using a facile ball milling preparation of B-nZVI/BC and PS.

## 1. Introduction

*p*-chloroaniline (PCA), as a type of chlorinated aromatic amine, is widely used as the main chemical raw material for pesticides, plastics, pigments, and pharmaceuticals [1,2]. PCA is a kind of persistent organic pollutants (POPs), which have environmental toxicity and will accumulate in the environment. A series of serious injuries to the blood system and nervous system (LD_50_ orally in rats: 0.31 g kg^−1^) were observed when humans were exposed to PCA [3]. Due to its environmental toxicity, adverse impacts on human health, and persistence, PCA has been listed as a priority pollutant by the US Environmental Protection Agency (EPA) and EU legislation [4]. For decades, the prolific use would inevitably give rise to the release of PCA into the soil. Therefore, much attention has been paid to exploring efficient technologies for the degradation of such a recalcitrant chlorinated aromatic compound [5,6]. Bioremediation was first utilized for PCA removal in soil, which included natural attenuation, bioaugmentation, biostimulation, and phytoremediation. Bioremediation relies on natural conditions through physical, chemical, and biological processes, while proper bio-stimulating agents are needed to stimulate indigenous microbial activities to enhance the degradation of the contaminant. Though biodegradation is relatively cheaper, the treatment is time-consuming and not suitable for situations with high concentrations of pollutants [7,8]. Physical methods such as thermal desorption and soil washing were also applied for PCA-contaminated soil remediation. Thermal desorption is a highly energy-consuming operation that will also cause the destruction of soil textures at high temperatures. Due to the high octanol/water (K_ow_) partition coefficient value of 1.83, PCA would strongly adsorb on the surface of soil particles and bond to soil organic matter. A large quantity of surfactant is required, which would decrease the effectiveness of the application and may lead to secondary pollution [9]. In addition, since PCA only transports from soil to other phases through physical methods, the indispensable follow-up treatment is still required.

Advanced oxidation processes (AOPs), which utilize oxidative free radicals, have become a promising technology for the remediation of recalcitrant organic pollutants in soil [10,11]. Persulfate (PS) is a strong oxidant with a redox potential (E_0_) of 2.01 V, which is higher than that of manganate (KMnO_4_, E_0_ = 1.70 V), hydrogen peroxide (H_2_O_2_, E_0_ = 1.78 V), and almost as effective as that of ozone (O_3_, E_0_ = 2.07 V). AOPs technology based on PS has received increasing attention [12]. More importantly, sulfate free radicals (SO_4_•^−^) with a higher oxidation potential (E_0_ = 2.60 V) can be generated after activation, which can accelerate the oxidative degradation of most organic pollutants into CO_2_ and H_2_O. Under neutral and alkaline conditions, hydroxyl radicals (•OH, E_0_ = 2.80 V) can also be produced by SO_4_•^−^. The transformed •OH may also be responsible for the oxidation of contaminants in conjunction with SO_4_•^−^ [13]. PS is a solid oxidant with the advantages of transport convenience and cost efficiency. Unlike H_2_O_2_, which is stable only under acidic conditions with a pH value lower than 5.0, PS can exist in soil stably for several weeks without activation. PS has become a potential oxidant widely applied in soil and groundwater treatment [14,15]. Though the addition of PS may have effects on soil physicochemical properties and microbial communities, the impact on the indigenous microbial community can be minimized with low doses of PS [16].

Heat, UV light, ultrasound, alkali, and transitional metals were utilized to activate PS. Carbonyl group, persistent free radicals (PFRs) and defect structures of carbon material could also active PS to generated SO_4_•^−^ in previous studies [17,18]. Among these, iron was a widely used activator for PS through Equations (1)–(3), as it was nontoxic, cheap, and naturally abundant in the environment. For example, Kang et al. [19] used ZVI-activating persulfate to degrade para-chloronitrobenzene (p-CNB) in soil, and the ZVI-persulfate system showed p-CNB removal of 88.7%. Wei et al. [20] studied the degradation of bentazon (BTZ) in the presence of ZVI and PS. Under the optimal concentrations of ZVI (4.477 mmol L^−1^) and PS (0.262 mmol L^−1^), 0.021 mmol L^−1^ BTZ was totally removed at an initial pH ≤ 7, and the BTZ removal well followed a pseudo-first-order kinetics pattern. However, degradation efficiency is low for the commercial ZVI because of its low specific surface area. To improve its reactivity, a successful alternative to nano-zero-valent iron particles (nZVI) has been constructed and proved its efficacy in environmental clean-up because of its small size, less than 100 nm. nZVI possessed the merits of a larger surface area, a high density of reactive surface sites, and ease of dispersion, which would significantly enhance the PS activation ability [21]. El-Temsah et al. [22] reported that nZVI was used to treat the DDT-contaminated soil (24 mg kg^−1^). The added nZVI led to about 50% degradation of DDT in spiked soil in 7 days. Yan et al. [23] revealed the nZVI/PS system was efficient for 1,2-dichlorobenzene (1,2-DCB)-contaminated soil remediation. The maximum degradation efficiency of 97.3% with total organic carbon (TOC) removal of 61.3% for 1,2-DCB with a contaminant level of 28.6 mg kg^−1^ in soil was achieved under the conditions of 67.2 mg L^−1^ nZVI, 1.2 mmol L^−1^ PS, and an initial pH of 7.5. For nZVI, it is easily agglomerated and oxidized in the air. Therefore, modified nZVI-based materials with changes in shape, size, and electron transfer reactions to obtain a stable structure and high specific surface area are expected [24,25].

2Fe^0^ + O_2_+ 2H_2_O → 2Fe^2+^ + 4OH^−^ (1)

Fe^0^ + 2H_2_O → Fe^2+^ + 2 OH^−^ + H_2_ (2)

Fe^2+^ + S_2_O_8_^2−^ → Fe^3+^ + SO_4_•^−^ + SO_4_^2−^ (3)

Selecting suitable templates as nZVI supporting materials on its surface was crucial to minimizing agglomeration and aerial oxidation. Porous materials, including inorganic materials and organic polymers with large specific surface areas, were especially popular for dispersion of nZVI by providing sufficient loading sites [26]. Carbon materials such as activated carbon (AC), carbon fiber, graphene, and graphene oxide (GO) with flexible structures, high surface areas, and considerable oxygen functionality would enhance the stability and reactivity of nZVI via complex formation [27,28,29]. In addition, the generated defects could alter the electron charge distribution on the carbon surface, providing the target-oriented physicochemical and electronic properties favored for contaminant adsorption and electron-transfer performance during PS activation processes [17]. It was known that conventional nZVI/carbon composites were produced using hazardous sodium borohydride (NaBH_4_) and other reducing agents or through a thermal reduction process. The above-mentioned reducing reagents are flammable, corrosive, and toxic, posing safety concerns for the environment [30]. The thermal reduction process incurs huge costs and generates toxic gases and waste streams. The application is intrinsically limited by the associated high cost and pollution concerns, and it is imperative to investigate cost-effective and sustainable green alternatives for large-scale production.

Mechanochemical surface functionalization of ball milling is a mature and clean process for producing high-activity solid powders. It was one of the most environmentally friendly, economically efficient, and promising methods that have been applied for sulfidated ZVI and bimetallic ZVI preparation [31,32]. During the ball milling process, the ZVI size and structure can be deformed by mechanical force, and the surface oxide layer will be crashed to expose the internal metallic iron core, which can effectively reduce the reaction activation energy and enhance chemical reactivity [33]. Additionally, due to the compaction effect between the solid particles, diffusion occurs through the solid-solid reaction, leading to surface modification, yielding novel characteristics and thus improving the reactivity of the composite [34,35]. Ball milling is a green method to synthesize iron-carbon composites with excellent properties for scale applications.

In this work, biochar-modified ZVI particles were synthesized through the ball milling method (B-nZVI/BC) and subsequently characterized as PS activators for the remediation of PCA in soil. Biochar is an emerging material widely used for hydrophobic organic compounds or heave metal adsorption removal because of its excellent porous structure, high specific surface area, and relative low cost [11]. Target contaminants will be conducive to pre-concentration, facilitating subsequent degradation. Functional groups and hybridized carbon structures also have a positive effect on PS activation due to electron transfer processes [36]. It is anticipated that biochar in B-nZVI/BC acts as both the supported material for nZVI and an activator for PS, and the synthesized B-nZVI/BC composite by the ball milling method could improve the performance for PS activation and thus increase the removal rate of PCA in contaminated soil. To our knowledge, little attention has been paid to the remediation of real PCA-contaminated soil from an industrial site. The main objectives of the present study were to assess the effectiveness of the remediation of PCA in soil with B-nZVI/BC and PS, explore the influence of B-nZVI/BC dosage and PS concentration on the degradation of PCA, identify the generated free radical species, and interpret the possible activation mechanism of B-nZVI/BC for PS. The intermediates of PCA were evaluated by a gas chromatographic mass spectrometer (GC-MS), and the possible degradation pathways of PCA were proposed in the B-nZVI/BC activated PS system.

## 2. Materials and Methods

### 2.1. Chemicals and Soils

PCA (C_6_H_6_ClN, >99.5%) was obtained from Sigma-Aldrich Co., Ltd. (St. Louis, MS, USA). Dichloromethane (>99.5%), ethyl acetate (>99.8%), and acetone (>99.5%) were purchased from Nanjing Chemical Reagent Co., Ltd. (Nanjing, China). Ethanol was obtained from Xilong Scientific Co., Ltd. (Shantou, China). PS (Na_2_S_2_O_8_, >98.0%) and commercial iron powder (ZVI, 400 mesh) were purchased from Sinopharm Chemical Reagent Co., Ltd. (Suzhou, China). 5,5-Dimethyl-1-pyrrolidine N-oxide (DMPO, >97.0%) and 2,2,6,6-tetramethyl-4-piperidinol (TEMP, 98%) were obtained from J&K Chemical (Shanghai, China). Sodium hydroxide (NaOH) and Hydrochloric acid (HCl) were received from Shanghai Lingfeng Chemical Reagent Co., Ltd. (Shanghai, China). All solutions were prepared with ultrapure water produced by the Millipore milli-Q system. PCA-contaminated soil was collected from a former pesticide manufacturing site in Nanjing, China. Soil samples were first sieved with a 2 mm mesh to remove gravel and stored for the subsequent experiments. The physical and chemical properties of soil are listed in Table 1.

### 2.2. Preparation of B-nZVI/BC

Biochar was produced from rice straw by the pyrolysis method in the first step, according to our previous work [11]. The rice straw was gathered in Nanjing, washed with ultrapure water several times, and dried in an oven at 70 °C for 12 h. The dried rice straw was then collected and placed in a muffle furnace for 6 h at a temperature of 500 °C under oxygen-limited conditions. After natural cooling, the produced biochar was washed with 1 mol L^−1^ HCl and then washed with deionized water to a neutral pH.

To prepare B-nZVI/BC, commercial iron powder and as-prepared biochar with a mass ratio of 1:1 were mixed and sealed in a jar (100 mL) with argon headspace. Zirconia balls with diameters of 5 mm, 6 mm, and 10 mm (7:2:1) were added into the jar, and then the milling was performed at 550 rpm for 12 h using a planetary ball mill. Every 15 min, ball milling was stopped for 5 min, then the rotation process was started in the opposite direction. After ball milling, the product of B-nZVI/BC was separated and collected.

### 2.3. Degradation Procedures

Sacrificial batch remediation experiments for soil were carried out in a 250-mL brown, sealed bottle. The PCA-contaminated soil was added to the bottle with a volume ratio of water to soil of 1:1. An appropriate amount of B-nZVI/BC and PS was added to the bottle consecutively. After that, the bottle was placed on a reciprocating shaker to initiate the reaction at a temperature of 25 ℃. Control tests were also conducted. At regular time intervals, the soil was sampled with the addition of ethanol to quench the reaction. PCA was subsequently extracted from the soil by sonicating in a water bath with extraction solvents that consist of acetone:dichloromethane:ethyl acetate (1:2:1, v:v:v). After centrifugation, PCA and its intermediates were quantitatively analyzed by Gas Chromatography-Mass Spectrometer (GC-MS). The details of the extraction process of PCA from soil are shown in Text S1. In the desorption and adsorption experiments, B-ZVI/BC was separated from the soil and subjected to oscillatory desorption with the extraction solvents. The concentrations of PCA in soil and adsorbed on B-nZVI/BC surfaces were detected separately. All the tests were performed in triplicate, from which the mean values were obtained.

### 2.4. Characterization Techniques

The crystalline phase pattern of B-nZVI/BC was analyzed with X-ray diffraction using Cu K_α_ radiation (XRD, X’TRA, Geneva, Swiss). The surface morphology and the elemental composition of the composite were measured by scanning electron microscopy (SEM, Hitachi S-4800, Tokyo, Japan) and transmission electron microscopy (TEM, FEI Tecnai G2 spirit, Eindhoven, Holland) equipped with energy dispersive spectroscopy (EDS, Bruker, QUANTAX 400, Saarland, Germany). The Brunauer-Emmett-Teller specific surface areas (SA_BET_) of B-nZVI/BC were observed using the N_2_ adsorption method (Micromeritics, ASAP 2020 M + C, Norcross, GA, USA). The surface compositions and the valence states of the composites were determined by X-ray photoelectron spectroscopy (XPS, Shimadzu, AXIS UltraDL, Kyoto, Japan). Reactive free radicals were recorded through an electron paramagnetic resonance (EPR) spectrometer (Bruker, EMX-10/12, Saarland, Germany).

### 2.5. Analytical Methods

The concentrations of PCA and its degradation intermediates were determined by GC-MS (Agilent, 7890A-5975C, Palo Alto, Santa Clara, CA, USA) with a HP-5 chromatographic column. The initial temperature of the GC oven was 35 °C and held for 2 min, which was then raised to 280 °C at a rate of 20 °C min^−1^. The flow rate of carrier gas (He) was 1.4 mL min^−1^, and the injection temperature was 220 °C [37]. The degradation intermediates of PCA in the B-nZVI/BC-PS system were derivatized with BSTFA/TCMS (99:1), which were also detected by GC-MS [38].

### 2.6. Statistical Analysis and Quality Assurance

Three parallel groups were set up for each batch experiment and presented as the mean ± standard error. The error bar was expressed by the standard deviation. Based on the changes in PCA concentration in the reaction process, the PCA degradation curves in all PS and PS activated systems were well fitted to the pseudo-first-order kinetic model of ln(*C*_t_/*C*_0_) = *k*t + b, where *C*_0_ and *C*_t_ are the concentrations of PCA in soil at time t = 0 and t, respectively, *k* is the apparent rate constant, t is the reaction time, and b is a constant of the pseudo-first-order rate constant in the degradation process [39]. Sample preparation and instrumental analysis methods were performed according to the EPA, including EPA/600/R-16/114 and EPA-8270E [40,41]. The laboratory control sample recovery (LCS) of PCA was 71.0%, and other quality control parameters are shown in Text S2. All data were compared by one-way analysis of variance (ANOVA) (*p* < 0.05) [42].

## 3. Results

### 3.1. Characterization of Prepared B-nZVI/BC

**Figure 1:**
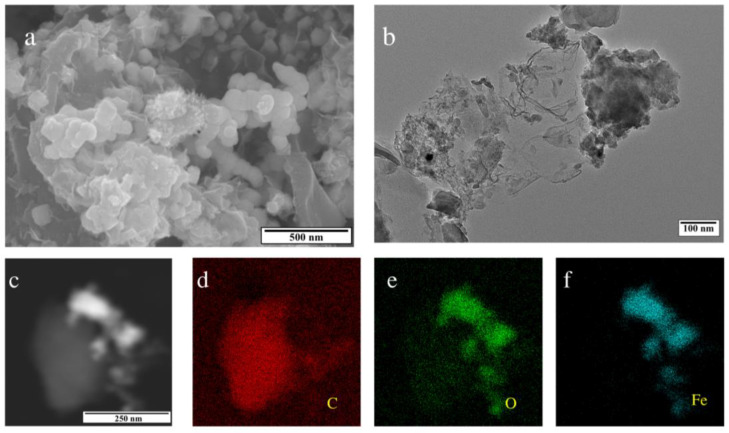
SEM image (**a**), TEM image (**b**), and TEM-EDS (**c**–**f**) images of B-nZVI/BC.

The morphological and chemical elemental composition analysis of the B-nZVI/BC was conducted, and the data are shown in Figure 1. The small size of the nanoscale spherical iron particles and lamellar cracks in the structured biochar were observed from the SEM image, which was probably due to the collision of the ball milling process [43]. In addition, spherical nZVI was homogenously distributed on the BC surface in Figure 1a, which was consistent with the observation of the TEM image in Figure 1b. TEM-EDS in Figure 1c–f showed that Fe, O, and C were the dominant elements of the prepared B-nZVI/BC, and the high coincidence of Fe and O elements indicated iron oxides might be formed due to the effect of oxidation in the air.

**Figure 2:**
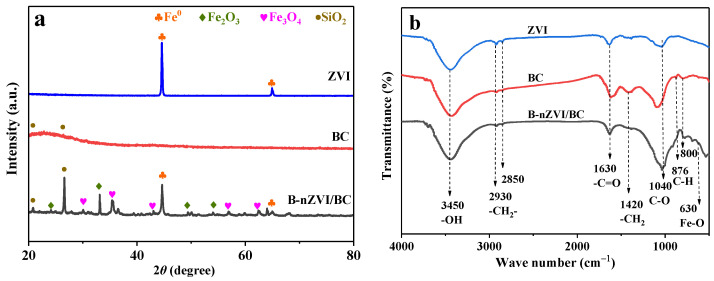

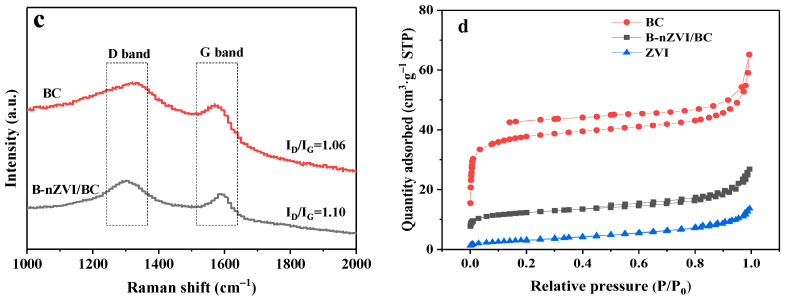
XRD patterns (**a**), FT-IR spectra (**b**), Raman spectra (**c**), and BET nitrogen adsorption-desorption isotherms (**d**) of ZVI, BC, and B-nZVI/BC.

The XRD patterns of ZVI, BC, and B-nZVI/BC are shown in Figure 2a. The diffraction peaks of ZVI at 2θ of 44.7° and 65.0° were indexed as the (110) and (200) crystal planes of α-Fe^0^ (JCPDS-99-0064). BC displayed an amorphous carbon with 2θ from 20° to 25° [40]. Both the peaks of amorphous carbon and α-Fe^0^ were observed in B-nZVI/BC, demonstrating the successful formation of α-Fe^0^ on BC surfaces [44]. The diffraction peaks at 2θ of 24.1°, 33.2°, 49.5°, and 54.1° ascribing to Fe_2_O_3_ (JCPDS-99-0060) and 30.1°, 35.4°, 43.1°, 57.0°, and 62.5° corresponding to Fe_3_O_4_ (JCPDS-99-0073) were due to the oxidation of B-nZVI/BC in the air, leading to the formation of iron oxides [45,46]. In addition, crystalline material with peaks at 20.9° and 26.6° can be indexed as SiO_2_ (JCPDS-99-0088), which is a constituent of rice straw biochar [47].

The FT-IR spectra are illustrated in Figure 2b. The peak at 3450 cm^−1^ was assigned to the stretching vibration of –OH [48]. Absorption bands at 2930 cm^−1^ and 2850 cm^−1^ were identified as the –CH_2_, and the peaks at 1630 cm^−1^ and 1040 cm^−1^ corresponded to aromatic C=O and C–O stretching vibrations, respectively [49]. After ball milling, a new peak at 630 cm^−1^ appeared, which can be assigned to the Fe-O-H bond between Fe and BC [50]. The results suggested the reaction between iron and the oxygen groups on the surface of the biochar occurred to form a uniformly loaded composite through a mechanical chemical reaction during the ball milling process.

The defective natures of BC and B-nZVI/BC were demonstrated by Raman spectroscopy in Figure 2c. Two major bands were illustrated, corresponding to the D band (~1300 cm^−1^) and the G band (~1570 cm^−1^) [51]. The G bond is closely related to the crystalline and graphitic structures; the D bond usually results from defects and distortions in the carbon layers. The ratio of the D band to the G band (I_D_/I_G_) can be implied as the defect degree of carbon materials [52]. The I_D_/I_G_ values were 1.06 and 1.10 for BC and B-nZVI/BC, respectively, indicating that the ball milling process was beneficial to increasing carbon defects. In addition, the G peak for BC was located at 1570 cm^−1^ and blue-shifted to 1590 cm^−1^ for B-nZVI/BC, suggesting that the charge was transferred from graphitic carbon to nZVI [53].

The N_2_ adsorption-desorption isotherms and porosity distributions of ZVI, BC, and B-nZVI/BC were illustrated in Figure 2d and Figure S1a. All of the adsorption-desorption isotherms were identified as type IV with a hysteresis loop, suggesting that mesopore structures (2 nm < pore size < 50 nm) existed in the prepared materials, which were further verified by the Barrett-Joyner-Halenda (BJH) pore size distribution. The pores in ZVI, BC, and B-nZVI/BC were distributed between 2 nm and 10 nm. From Figure S1b–d (in upplementary Materials), the average particle size of B-nZVI/BC was smaller, and the distribution was more uniform compared with raw ZVI and BC, which was due to the collision through the ball milling process. The SA_BET_ of ZVI, BC, and B-nZVI/BC were 11.9, 41.0, and 116.2 m^2^ g^−1^, respectively (Table S1).

### 3.2. PCA Degradation Kinetics in the B-nZVI/BC-PS System

**Figure 3:**
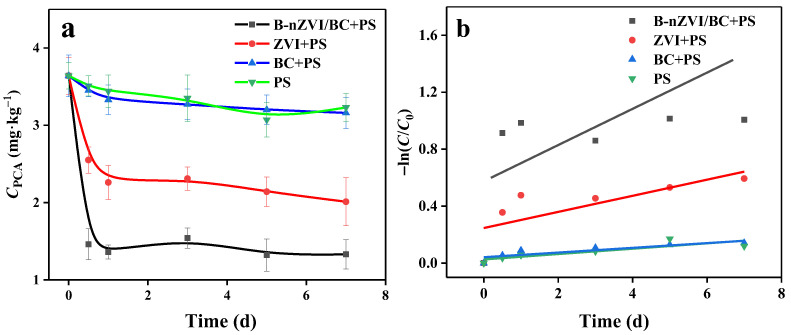
Kinetics curves of the PCA removal (**a**) and pseudo-first-order kinetics of PCA in ZVI, BC, and B-nZVI/BC activated PS systems (**b**). Reaction conditions: [PS]_0_ = 42.0 mmol L^−1^, [B-nZVI/BC]_0_ = 4.8 g kg^−1^, [ZVI]_0_ = 2.4 g kg^−1^, [BC]_0_ = 2.4 g kg^−1^, pH_0_ = 7.49, and T = 25 °C.

The removal of PCA with PS activated by ZVI, BC, and B-nZVI/BC was conducted, and the kinetic data are shown in Figure 3a. A control experiment in the absence of activators and PS was first carried out, which showed less than 5% PCA loss under the tested conditions after 7 d. With the addition of PS, the removal efficiency of PCA was 11.26%, which might be due to the activation of PS by iron-manganese compounds and organic matter in soil [10]. BC resulted in a PCA removal efficiency of 13.18% with the effect of adsorption and the weak activation property of carbon, and the presence of ZVI was attributed to a PCA removal efficiency of 44.78%. However, in the B-nZVI/BC-PS system, the concentration of PCA in soil was dramatically decreased from 3.64 mg kg^−1^ to 1.33 mg kg^−1^ with the addition of 4.8 g kg^−1^ B-nZVI/BC and 42.0 mmol L^−1^ PS, corresponding to a PCA removal efficiency of 63.46%. The concentration of PCA after reaction was much lower than the calculated remediation target value of 1.96 mg kg^−1^ for soil.

As shown in Figure 3b, the PCA degradation curves in all PS and PS-activated systems well-fitted the pseudo-first-order kinetic model. From Figure 3b, the *k* value for PCA degradation with PS was 0.019 d^−1^, and the *k* values for PCA degradation in BC-PS and ZVI-PS systems were 0.017 d^−1^ and 0.057 d^−1^, respectively. In the B-nZVI/BC-PS system, the first-order kinetic rate constant of *k* was 0.077 d^−1^, which was 4.5 times that in the BC-PS system and almost 1.4 times that in the ZVI-PS system (Table S2), highlighting the synergistic effect of the ball milling process on the reactivity of prepared B-nZVI/BC. The ball milling process decreased the size of ZVI (observed from the characterization results in Figure S1d) and significantly enhanced the defect and porous structures of prepared B-nZVI/BC, which comprehensively improved the activation capability towards PS and increased the PCA degradation and *k* value.

To explore the effect of adsorption in the B-nZVI/BC-PS oxidation system, the amount of adsorbed PCA on the surface of B-nZVI/BC was desorbed after a 7 d reaction; the removal rate of PCA was 63.46%, and only 1.64% of PCA was adsorbed on the surface of B-nZVI/BC (Figure S2a), indicating that most PCA was degraded by oxidation. In the B-nZVI/BC system without PS, the removal rate of PCA adsorbed on the B-nZVI/BC surface was only 5.22% (Figure S2b). It can be concluded that oxidation degradation played a major role in PCA removal in the system of B-nZVI/BC-PS.

### 3.3. Effect of Reaction Parameters on the Degradation of PCA

**Figure 4:**
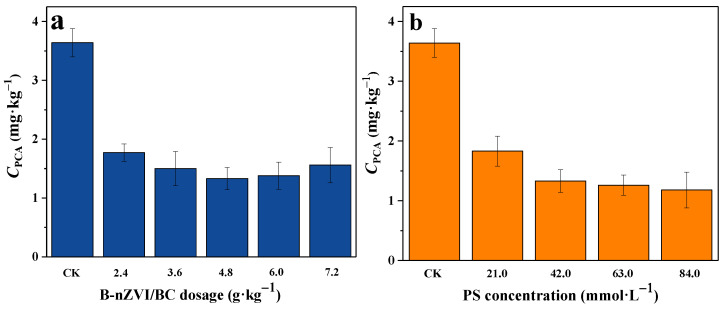
Effects of B-nZVI/BC dosage (**a**) and PS concentration (**b**) on the degradation of PCA in the B-nZVI/BC-PS system. Reaction conditions: [PS]_0_ = 42.0 mmol L^−1^ for (**a**), [B-nZVI/BC]_0_ = 4.8 g kg^−1^ for (**b**), pH_0_ = 7.49, and T = 25 °C.

The effect of reaction parameters on the degradation of PCA was studied, and the data are shown in Figure 4. B-nZVI/BC is the intrinsic driving force for PS activation to generate free radicals, and the dosage will have a great influence on PCA degradation. When the dosage of B-nZVI/BC increased from 2.4 g kg^−1^ to 4.8 g kg^−1^, the concentration of PCA in soil decreased from 1.77 mg kg^−1^ to 1.33 mg kg^−1^ correspondingly. Further increasing the dosage of B-nZVI/BC to 6.0 g kg^−1^ and 7.2 g kg^−1^, the concentration of PCA in soil after the reaction increased to 1.41 mg kg^−1^ and 1.56 mg kg^−1^, respectively. Zero-valent iron could be acted as an alternative source of Fe^2+^ for PS activation under aerobic and anaerobic conditions [54]. When a small amount of B-nZVI/BC was added, SO_4_•^−^ was produced and accounted for PCA degradation immediately, leading to a fast decrease of PCA in soil. However, when B-nZVI/BC were overdosed, too many SO_4_•^−^ radicals were generated instantaneously, and the quenching reaction between excess Fe^2+^ and SO_4_•^−^ occurred and directly consumed the generated SO_4_•^−^ through Equation (4) [55]. Thus, considerable disappearance of SO_4_•^−^ radicals was proceeded, and decreased degradation efficiency of PCA was observed.

Fe^2+^ + SO_4_•^−^ → Fe^3+^ + SO_4_^2−^ (4)

Figure 4b shows the effects of the concentrations of PS on the degradation of PCA. SO_4_•^−^ is produced by the decomposition of PS; increasing PS concentration would promote the amount of SO_4_•^−^ and hence improve PCA degradation. The concentration of PCA in soil rapidly decreased to 1.83 mg kg^−1^ and 1.33 mg kg^−1^ when the concentration of PS was increased from 21.0 mmol L^−1^ to 42.0 mmol L^−1^ in the presence of 4.8 g kg^−1^ B-nZVI/BC. With the same amount of activator, a relative higher PS concentration accounted for a greater amount of generated SO_4_•^−^ radicals and thus resulted in an efficient degradation of PCA. Further increasing the PS concentration to 63.0 mmol L^−1^ and 84.0 mmol L^−1^, the concentration of PCA in soil slightly decreased to 1.28 mg kg^−1^ and 1.18 mg kg^−1^. PS was activated by B-nZVI/BC, and the influence of PS concentration on PCA degradation was inconspicuous in the presence of excess PS with a given amount of B-nZVI/BC. Thus, no significant increase in PCA degradation efficiency was observed when the concentration of PS was beyond 42.0 mmol L^−1^, corresponding to the B-nZVI/BC to PS mass ratio of 1:2. It should be noted that under the optimum mass ratio condition with the addition of 14.4 g kg^−1^ B-nZVI/BC and 126.0 mmol L^−1^ PS, the concentration of PCA decreased to as low as 0.15 mg kg^−1^, corresponding to a degradation efficiency of 95.9% (Figure S3).

### 3.4. Possible Activation Mechanism of PS by B-nZVI/BC

#### 3.4.1. XPS Analysis before and after Reaction

**Figure 5:**
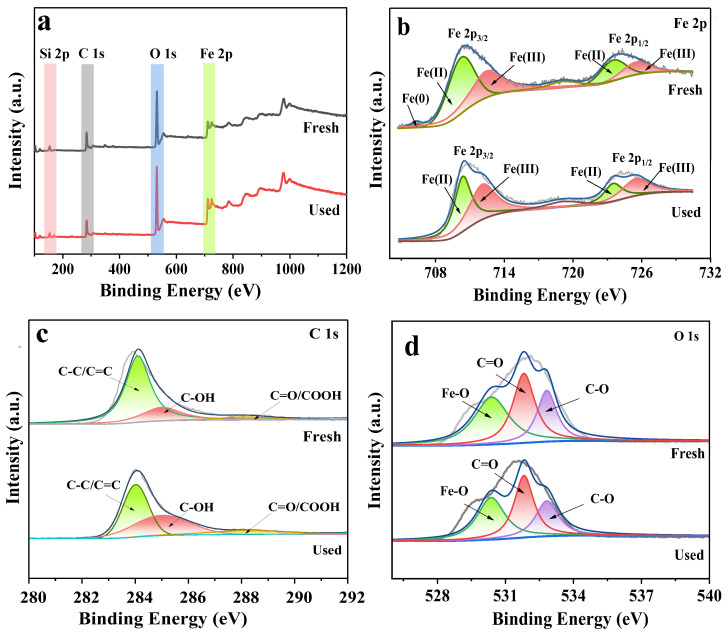
XPS survey spectra (**a**), Fe 2p (**b**), C 1s (**c**), and O 1s (**d**) spectra of B-nZVI/BC before and after reaction.

To explore the possible activation mechanism of PS by B-nZVI/BC, XPS characterization was utilized to determine the chemical status and chemical composition of B-nZVI/BC before and after the reaction. Compared with fresh B-nZVI/BC, the atomic percentage of O increased from 47.29% to 50.42%, and the percentages of C and Fe decreased from 29.4% to 25.5% and from 6.44% to 6.42%, respectively, after the reaction (Figure 5a and Figure S4). The increased O content suggested that the B-nZVI/BC was oxidized during the PS activation process, and the decreased C and Fe content indicated that C and Fe participated in the PS activation. After the reaction, it was also found that the surface of B-nZVI/BC became rough (Figure S5).

The Fe 2p spectra of the B-nZVI/BC in Figure 5b showed the spin-orbit doublets of Fe 2p_1/2_ and Fe 2p_3/2_ peaks. The small peak at 706.3 eV was ascribed to Fe(0) [56]. The peaks at 710.3 eV and 723.4 eV belonged to Fe(II), and the peaks at 712.8 eV and 725.6 eV were attributed to Fe(III). The peak at 719.1 eV was the satellite peak of Fe 2p. The existence of the Fe(0) peak proved that zero-valent iron was formed in B-nZVI/BC, and it is inevitably oxidized during storage and analysis, resulting in the appearance of Fe(II) and Fe(III) [40,57,58]. After activation, the peak of Fe(0) was not detected, hinting that the transformation of Fe(0) to Fe(II) or Fe(III) on the surface of B-nZVI/BC occurred. The ratio of Fe(II) decreasing from 51.1% to 44.2% and Fe(III) increasing from 41.1% to 48.5% (Table S3) was due to the electron transfer from Fe(II) to PS to induce the activation of PS, and Fe(III) was formed on the surface of B-nZVI/BC [52]. The peak strength of ferric iron oxide increased and zero-valent iron decreased after the reaction in XRD characterization (Figure S6), which also verified the processes.

Figure 5c illustrates the C1s spectra of B-nZVI/BC. The peaks at 284.0 eV, 285.0 eV, and 287.8 eV corresponded to C=C/C–C, C–OH and C=O/COOH, respectively [59]. After reaction, the percentage of C=C/C–C decreased from 66.48% to 42.98%, while the percentage of C-OH increased from 20.87% to 32.43%, and the percentage of C=O/COOH increased from 5.93% to 16.23% (Table S4). The peaks at 284.0 eV and 285.0 eV corresponded to *sp*^2^ and *sp*^3^ hybridized carbons; the electron migrated from graphitic *sp*^2^ hybridized carbon to PS, leading to a decreased percentage of C=C/C–C. The graphitic *sp*^2^ hybridized carbon structure (C=C) was oxidized, accounting for the formation of oxygen functional groups and *sp*^3^ hybridized carbon (i.e., C–OH and C=O/COOH) in the PS activation process [40,60]. The decrease in C=C/C–C, along with the increase in C–OH and C=O/COOH suggested that graphitic carbon was the reactive site for PS activation. As shown in Figure 5d, the O 1s peaks at 530.4 eV, 531.8 eV, and 532.8 eV of B-nZVI/BC belonged to Fe–O, C–OH, and C=O [61]. The Fe–O peak proved the existence of iron oxide. Similarly, compared with fresh B-nZVI/BC, the percentages of both C–OH and C=O increased after the reaction (Table S5 and Figure S7). The TEM-EDS (Figure S8) also showed that the percentage of element O increased from 8.14% to 39.63% after the reaction.

#### 3.4.2. Identification of Reactive Oxygen Species

**Figure 6:**
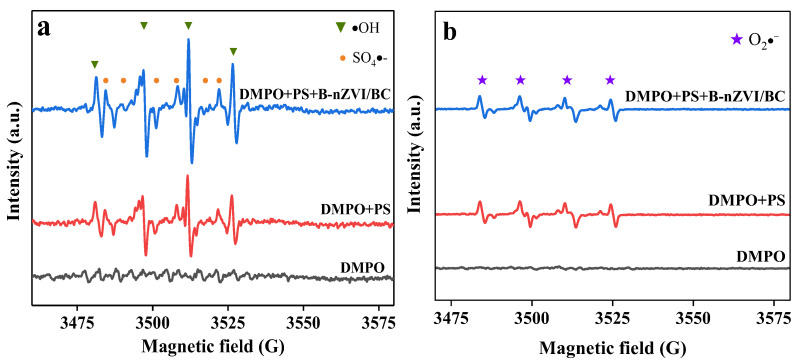
EPR measurements for SO_4_•^−^, •OH (**a**), and O_2_•^−^ (the EPR spectrometric detection of O_2_•^−^ was performed in DMSO solution, which was designed to avoid the influence of SO_4_•^−^ and •OH) (**b**) in various activated PS systems. Reaction conditions: [PS]_0_ = 42.0 mmol L^−1^, [DMSO]: [H_2_O] =9:1 (volume ratio), [DMPO] = 200.0 mmol L^−1^, [B-nZVI/BC]_0_ = 4.8 g kg^−1^, pH_0_ = 7.49, and T = 25 °C.

Reactive oxygen species (ROSs) such as SO_4_•^−^, •OH, O_2_•^−^, and ^1^O_2_ might exist in the metal/carbon/PS systems in previous studies [62]. In order to identify the ROSs species, EPR tests were performed, and the data are shown in Figure 6. DMPO was used as a spin-trapping reagent for SO_4_•^−^, •OH, and O_2_•^−^, and TEMP was used to capture ^1^O_2_ [63,64]. No signals were detected when only trapping agents were added to the soil. While weak signals of DMPO-SO_4_•^−^ (six lines of 1:1:1:1:1:1), DMPO-•OH (four lines of 1:2:2:1), and DMPO-O_2_•^−^ (four lines of 1:1:1:1) adducts appeared in the soil/PS/DMPO system, corresponding to the natural present iron oxide and dissolved Fe from iron-containing minerals for PS activation [65].

After adding B-nZVI/BC, the signals of DMPO-SO_4_•^−^ and DMPO-•OH adducts were obviously enhanced and observed, and the signal of DMPO-O_2_•^−^ adduct was also detected, indicating that the B-nZVI/BC-activated PS reaction occurred rapidly to promote the formation of SO_4_•^−^, •OH, and O_2_•^−^. For B-nZVI/BC, Fe^2+^ was formed under aerobic and anaerobic conditions in accordance with Equations (1) and (2), and subsequently for PS activation to generate SO_4_•^−^ (Equation (3)). Besides, BC also activated PS to generate SO_4_•^−^ due to electron transfer from graphitic *sp*^2^ hybridized carbon to PS. Additionally, •OH could be generated by the SO_4_•^−^ transformation with the reaction of OH^−^ and H_2_O, as shown in Equations (5) and (6) [66]. With the reaction between PS and OH^−^/H_2_O, HO_2_^−^ was formed and subsequently reacted with PS to generate O_2_•^−^ from Equations (7) and (8). Signals of DMPO-^1^O_2_ with three lines of 1:1:1 [62] were not found in the presence of ZVI, BC, and B-nZVI/BC activated PS systems, suggesting that the non-radical species of ^1^O_2_ was not generated and participated in the degradation of PCA (Figure S9a,b).

SO_4_•^−^ + OH^−^ → SO_4_^2−^ + •OH (5)

SO_4_•^−^ + H_2_O→ SO_4_^2−^ + •OH + H^+^ (6)

S_2_O_8_^2−^ + 2H_2_O (OH^−^) → HO_2_^−^ + 2SO_4_^2−^ + 3H^+^ (7)

HO_2_^−^ + S_2_O_8_^2−^ → SO_4_•^−^ + SO_4_^2−^ + H^+^ + O_2_•^−^ (8)

As illustrated in Figure S9c, the signal intensities of free radical adducts varied in different reaction systems. The peaks of DMPO-SO_4_•^−^ and DMPO-•OH in the B-nZVI/BC-PS system were the highest, followed by ZVI and BC. However, the intensity of the DMPO-O_2_•^−^ adduct in the presence of B-nZVI/BC was slightly stronger than that without any activator (Figure S9d). The order of radical intensity was in accordance with the PCA degradation ratio and the apparent rate constants illustrated in Figure 3b, indicating that SO_4_•^−^ and •OH were the dominant ROSs accounting for PCA degradation. Besides, the redox potential of O_2_•^−^ was 2.4 V, which could also participate in PCA degradation [67].

Based on the above analyses, iron-induced and carbon-induced activation of PS contributed to the efficient removal of PCA with B-nZVI/BC. Both the electron migration from nZVI to PS and the electron transfer from graphitic *sp*^2^ hybridized carbon to PS generated SO_4_•^−^, •OH, and O_2_•^−^. In the process, Fe(0) was transformed into Fe(II) and Fe(III), and the produced Fe(II) could activate PS to generate SO_4_•^−^. The produced SO_4_•^−^ was transformed into •OH, and O_2_•^−^ was generated from the reaction between PS and OH^−^/H_2_O. Additionally, the electron transfer from *sp*^2^ hybridized carbon structures to PS also promoted the generation of free radicals. In summary, the electron transport processes of nZVI and carbon structures in B-nZVI/BC accounted for PS activation, resulting in SO_4_•^−^, •OH, and O_2_•^−^ formation and subsequently PCA degradation in soil.

### 3.5. Degradation Pathways of PCA

In an oxidative system for the degradation of PCA, cationic radicals were first generated and further oxidized to release chloride ions and/or ammonium ions. The intermediates were decomposed into small molecular organic acids or alcohols after the benzene ring was opened [68]. In order to explore the degradation pathways of PCA, derivatization experiments were carried out with silanization reagents to detect the intermediate products by GC-MS. As shown in Figure S10, six organic intermediates were identified. 1-chlorobutane (t_R_ = 3.501 min, *m/z* = 92.6) and the derivatized product of glycine (t_R_ = 14.173 min, *m/z* = 165.3) were detected, which might be caused by carbon chain breakage after the benzene ring of PCA was opened. Other derivatives of organic acids and alcohols with four carbon atoms were also measured, including the derivatized products of butanol (t_R_ = 23.990 min, *m/z* = 104.2) and butanol acid (t_R_ = 7.650 min, *m/z* = 118.2). Derivatives of small organic acids or alcohols with two carbon atoms, including ethylene glycol (t_R_ = 9.774 min, *m/z* = 122.3) and glycolic acid (t_R_ = 14.087 min, *m/z* = 136.3), were also determined, which might be due to the chain scission of long-chain carbon-containing organics.

**Figure 7:**
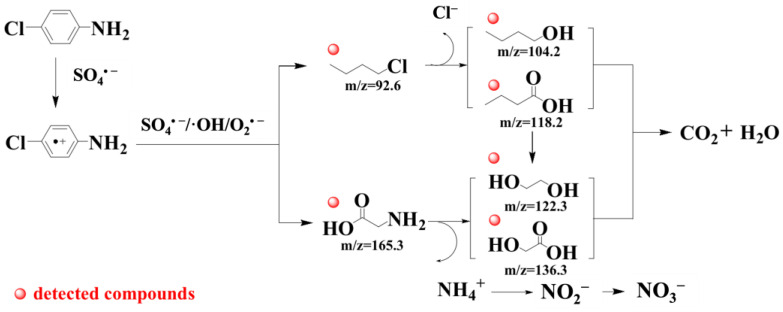
Degradation pathways of PCA in the B-nZVI/BC-PS system.

Based on the above results, the degradation pathways of PCA are proposed in Figure 7. SO_4_•^−^ was generated in the B-nZVI/BC activated PS system, which would also react with H_2_O/OH^−^ to form •OH. O_2_•^−^ was also generated by the effects of PS and H_2_O/OH^−^. SO_4_•^−^ attacked PCA to form PCA radical cation (PCA•^+^) first through the action of charge transfer. Secondly, the ring of PCA•^+^ was opened to form 1-chlorobutane and glycine due to the co-effect of SO_4_•^−^, •OH, and O_2_•^−^. 1-chlorobutane was oxidized into butanol or butyric acid subsequently. The oxidation of ROSs on PCA might lead to the production of Cl^−^ and chlorine-containing inorganic ions as well [6]. The glycine could be deaminated to form ammonium ions (NH_4_^+^), which were oxidized into nitrite ions (NO_2_^−^) and eventually nitrate ions (NO_3_^−^). While the remainder was oxidized into ethylene glycol and glycolic acid. The carbon chains of butanol and butyric acid could also be broken to form ethylene glycol and glycolic acid. Finally, the above-mentioned small molecular organic acids and alcohols were completely mineralized into CO_2_ and H_2_O.

## 4. Conclusions

B-nZVI/BC was successfully fabricated through ball milling and applied for PS activation to decompose PCA in soil. The concentration of PCA after remediation was much lower than the remediation target value of 1.96 mg kg^−1^, and the degradation efficiency of 95.9% was achieved in the presence of 14.6 g kg^−1^ B-nZVI/BC and 126.0 mmol L^−1^ PS under initial pH 7.49. Free radicals of SO_4_•^−^, •OH, and O_2_•^−^ generated from the redox effect of nZVI and the electron transfer processes through the *sp*^2^ hybridized carbon structure of biochar were identified and accounted for in PCA degradation. Six major degradation intermediates of 1-chlorobutane, glycine, butanol, butanol acid, ethylene glycol, and glycolic acid were measured, and the degradation pathways of PCA were proposed. This study gives a new insight into the mechanism of the reactivity of B-nZVI/BC for PS, providing a successive and effective process for the remediation of PCA-contaminated soil.

## Figures and Tables

**Table 1:** The physical and chemical properties of soil.

Lithology	Moisture (%)	Dry Density (g cm^−3^)	Specific Gravity (g cm^−3^)	Organic Matter (g kg^−1^)	pH	PCA (mg kg^−1^)
silty clay	22.8 ± 1.2	1.67 ± 0.07	2.71 ± 0.05	1.9 ± 0.02	7.49 ± 0.4	3.64 ± 0.19

## Data Availability

Not applicable.

## Supplementary Materials

The following supporting information can be downloaded at: https://www.mdpi.com/article/10.3390/nano13091517/s1. Text S1: Extraction process and detection method of PCA in soil; Table S1: BET-N_2_ specific surface areas and pore volumes of ZVI, BC and B-nZVI/BC; Table S2: Parameters of pseudo-first-order kinetic model of for PCA degradation in the systems of B-nZVI/BC-PS, ZVI-PS, BC-PS and PS; Table S3: Percentages of iron species of B-nZVI/BC before and after reaction in Fe 2p XPS spectra; Table S4: Percentages of carbon groups of B-nZVI/BC before and after reaction in C 1s XPS spectra; Table S5: Percentages of oxygen groups of B-nZVI/BC before and after reaction in O 1s XPS spectra; Figure S1: Pore size distribution of ZVI, BC and B-nZVI/BC (a), particle hydrodynamic diameter distribution of ZVI (b), BC (c) and B-nZVI/BC (d); Figure S2: Kinetics curves of the PCA removal in the B-nZVI/BC-PS system (a) and B-nZVI/BC system without PS (b). Reaction conditions: [PS]_0_ = 42.0 mmol L^−1^ in Figure S2a; [B-nZVI/BC]_0_ = 4.8 g kg^−1^, pH_0_ = 7.49, and T = 25 °C; Figure S3: PCA removal with different ratios of PS and B-nZVI/BC. Reaction conditions of 1: [PS]_0_ = 84.0 mmol L^−1^, [B-nZVI/BC]_0_ = 9.6 g kg^−1^, pH_0_ = 7.49 and T = 25 °C; reaction conditions of 2: [PS]_0_ = 126.0 mmol L^−1^, [B-nZVI/BC]_0_ = 14.4 g kg^−1^, pH_0_ = 7.49 and T = 25 °C; Figure S4: Atom Percentages of B-nZVI/BC before and after reaction in the XPS survey spectra; Figure S5: SEM of B-nZVI/BC before (a) and after reaction (b); Figure S6: XRD patterns of B-nZVI/BC after reaction; Figure S7: FT-IR of B-nZVI/BC after reaction; Figure S8: TEM-EDS of B-nZVI/BC before (a) and after reaction (b); Figure S9: EPR measurements for verify the radicals of ^1^O_2_ (a), ^1^O_2_ (b), SO_4_•^−^, •OH (c) and O_2_•^−^ (The EPR spectrometric detection of O_2_•^−^ was performed in DMSO solution, which was designed to avoid the influence of SO_4_•^−^ and •OH) (d) in systems of B-nZVI/BC-PS, BC-PS, and ZVI-PS. Reaction conditions: [PS]_0_ = 42.0 mmol L^−1^, [DMSO]: [H_2_O] =9:1 (volume ratio), [DMPO] = [TEMP] = 200.0 mmol L^−1^, [B-nZVI/BC]_0_ = 4.8 g kg^−1^, [ZVI]_0_ = 2.4 g kg^−1^, [BC]_0_ = 2.4 g kg^−1^, pH_0_ = 7.49, and T = 25 °C; Figure S10: GC-MS ion spectra at the reaction time of 5 min (a), 30 min (b), and 120 min (c) and its derivatives from BSTFA-silylated degradation intermediates (d-i) of PCA in the B-nZVI/BC-PS system.
