# Supplementary material for: Efficient Remediation of p-chloroaniline Contaminated Soil by Activated Persulfate Using Ball Milling Nanosized Zero Valent Iron/Biochar Composite: Performance and Mechanisms
Source: Nanomaterials (Basel). 2023 Apr 29;13(9):1517. doi: 10.3390/nano13091517 (PMC10180579; doi:10.3390/nano13091517)
Supplement: Supplementary file 1 [file nanomaterials-13-01517-s001.zip › nanomaterials-2361612-supplementary.pdf]

Supplementary Materials

Efficient Remediation of *p*-chloroaniline Contaminated Soil by Activated Persulfate Using Ball Milling Nanosized Zero Valent Iron/Biochar Composite: Performance and Mechanisms

Zihan Guo [1,2], Dong Wang [3], Zichen Yan [1,2], Linbo Qian [1,2], Lei Yang [1,2], Jingchun Yan [1,2,*] and Mengfang Chen [1,2]

1 Key Laboratory of Soil Environment and Pollution Remediation, Institute of Soil Science, Chinese Academy of Sciences; Nanjing 210008, China
2 University of Chinese Academy of Sciences, Beijing 100049, China
3 Jiangsu Environmental Engineering Technology Co., Ltd., Nanjing 210019, China
* Correspondence: jcyan@issas.ac.cn; Tel.: +86−25−8688−1848

Text S1. Extraction process and detection method of PCA in soil

The collected soil was mixed thoroughly, discard any foreign objects such as sticks, leaves, and rocks. After freeze-dried, weighted 10.0 g soil into the tared extraction tube, then added 15 mL extraction solvents which was consist of acetone: dichloromethane: ethyl acetate (1:2:1, v:v:v). Extracted the soil by sonicating in a water bath for 1h. Then centrifuged for 2 minutes at 2000 rpm. Extracted the soil twice more by adding approximately 10 mL of the extraction solvent. The combined extract concentrates were subjected to purification processed in a magnesium silicate column. Evaporated the solvent volume to just below 1 mL by blowing a gentle stream of clean dry nitrogen above the extract. The internal wall of the concentrator tube rinsed down several times with dichloromethane. Adjusted the final volume to 1.0 mL with dichloromethane and analyzed the PCA by gas chromatographic mass spectrometer (GC-MS). All the tests were performed in triplicate, by which the mean values were obtained.

Text S2 Analytical quality control information

Three parallel experiments were carried out to ensure and control the quality of the analysis program. Sample preparation and instrumental analysis methods were performed according to the United States Environmental Protection Agency (EPA) including EPA/600/R-16/114 and EPA-8270E [1-2].

The removal rate was represented by the average value of the three parallel experiments, and the error bar was expressed by standard deviation and calculated by the formula of $S=\sqrt{\frac{\sum_{i=1}^{n}(x_i-\bar{x})^2}{n-1}}$. Where S represents standard deviation, n is the number of values, i is the ith value, and $\bar{x}$ represents the average value.

The recovery rate of PCA by the extraction method was given in the manuscript. The detection limit was 0.09 $mg^{-1}\cdot kg^{-1}$, the matrix sample recovery (MS) and laboratory control sample recovery (LCS) of PCA were 57.0% and 71.0% respectively, which met the requirement of EPA (50%-150%) [1-3].

Table S1. BET-N_2 specific surface areas and pore volumes of ZVI, BC and B-nZVI/BC.

Material	SA_{BET} ($m^2\cdot g^{-1}$)	Pore volume[a] ($cm^3\cdot g^{-1}$)
ZVI	11.9	0.0155
BC	116.2	0.0760
B-nZVI/BC	41.0	0.0317

[a] Single point adsorption total pore volume of pores less than 40.4123 nm diameter at P/P_0 = 0.95.

Table S2. Parameters of pseudo-first-order kinetic model of for PCA degradation in the systems of B-nZVI/BC-PS, ZVI-PS, BC-PS and PS.

Reaction system	c_0 (mg/kg)	c_t (mg/kg)	Removal efficiency	k (d^{-1})
B-nZVI/BC-PS	3.64±0.24	1.33±0.19	63.46%±0.07	0.077
ZVI-PS	3.64±0.24	2.01±0.31	44.78%±0.12	0.057
BC-PS	3.64±0.27	3.16±0.2	13.19%±0.08	0.017
PS	3.64±0.17	3.23±0.18	11.26%±0.07	0.019

Table S3. Percentages of iron species of B-nZVI/BC before and after reaction in Fe 2p XPS spectra.

Iron state	Before reaction	After reaction
Fe(0)	1.2%	0
Fe(II)	51.1%	44.2%
Fe(III)	41.1%	48.5%

Table S4. Percentages of carbon groups of B-nZVI/BC before and after reaction in C 1s XPS spectra.

Carbon groups	Before reaction	After reaction
C–C/C=C	66.48%	42.98%
C–OH	20.87%	32.43%
C=O/COOH	5.93%	16.23%

Table S5. Percentages of oxygen groups of B-nZVI/BC before and after reaction in O 1s XPS spectra.

Oxygen groups	Before reaction	After reaction
Fe–O	39.54%	36.69%
C–OH	36.52%	38.01%
C=O	23.94%	25.29%

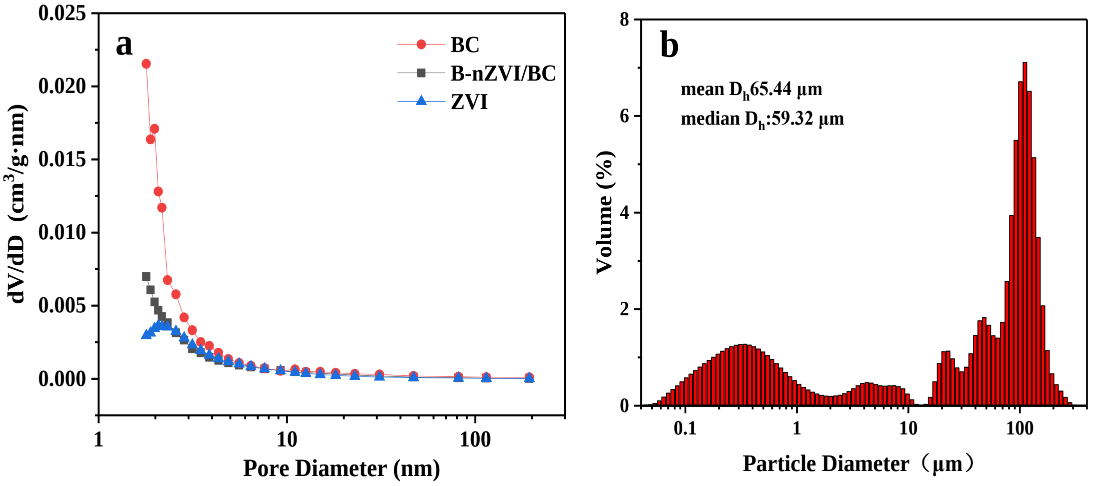

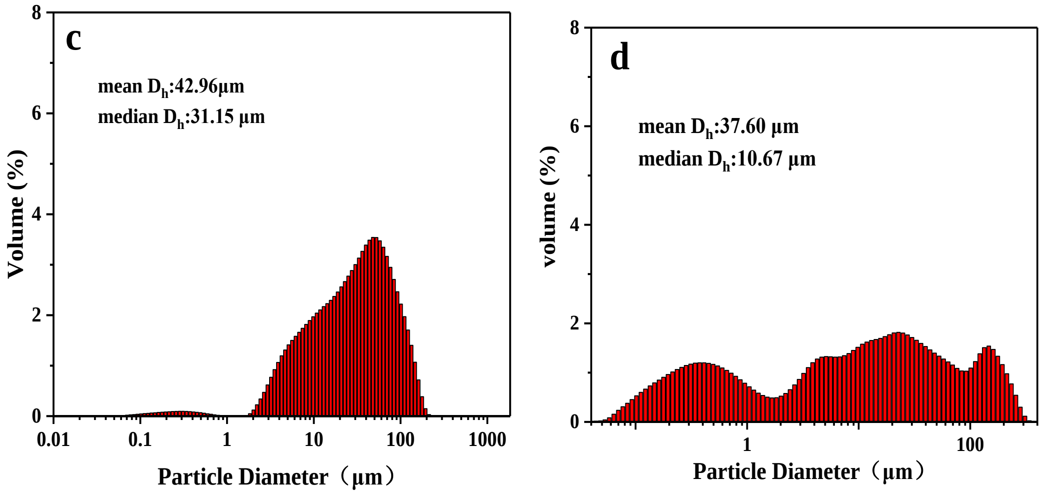

Figure S1. Pore size distribution of ZVI, BC and B-nZVI/BC (**a**), particle hydrodynamic diameter distribution of ZVI (**b**), BC (**c**) and B-nZVI/BC (**d**).

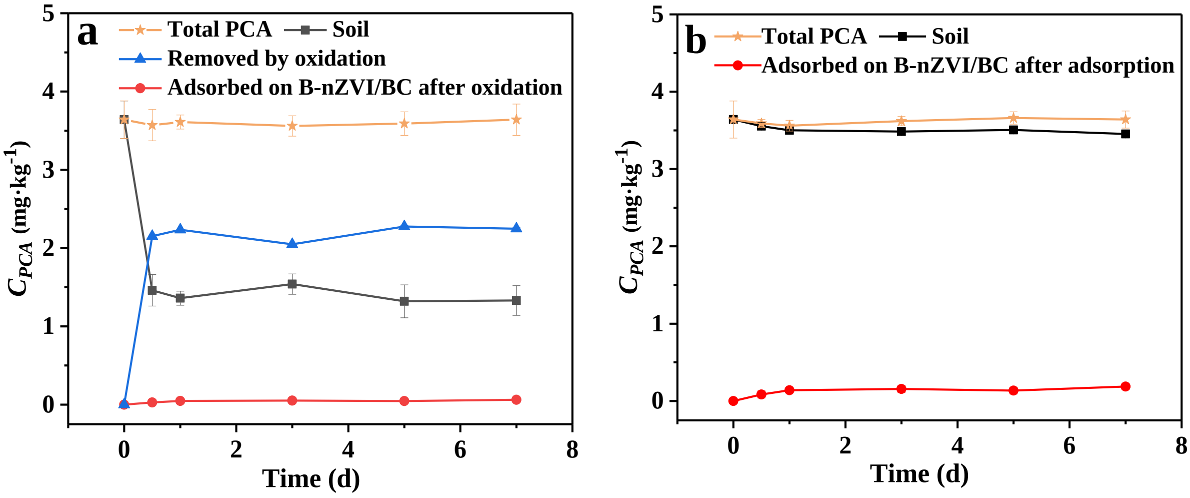

Figure S2. Kinetics curves of the PCA removal in the B-nZVI/BC-PS system (**a**) and B-nZVI/BC system without PS (**b**). Reaction conditions: $[PS]_0 = 42.0$ mmol L^{-1} in Figure S2a, $[\text{B-nZVI/BC}]_0 = 4.8$ g kg^{-1}, $pH_0 = 7.49$ and T = 25 °C.

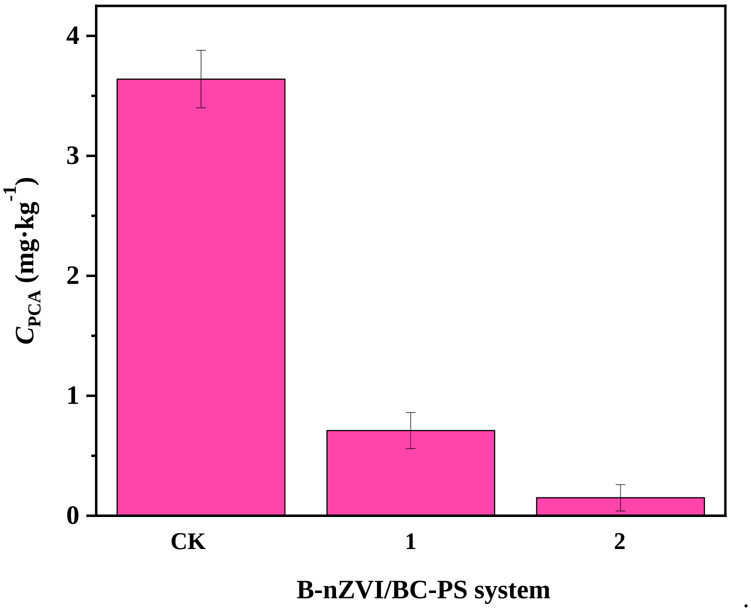

Figure S3. PCA removal with different ratio of PS and B-nZVI/BC. Reaction conditions of 1: $[PS]_0$ = 84.0 mmol L^{-1}, $[B\text{-}nZVI/BC]_0$ = 9.6 g kg^{-1}, pH_0 = 7.49 and T = 25 °C; Reaction conditions of 2: $[PS]_0$ = 126.0 mmol L^{-1}, $[B\text{-}nZVI/BC]_0$ = 14.4 g kg^{-1}, pH_0 = 7.49 and T = 25 °C.

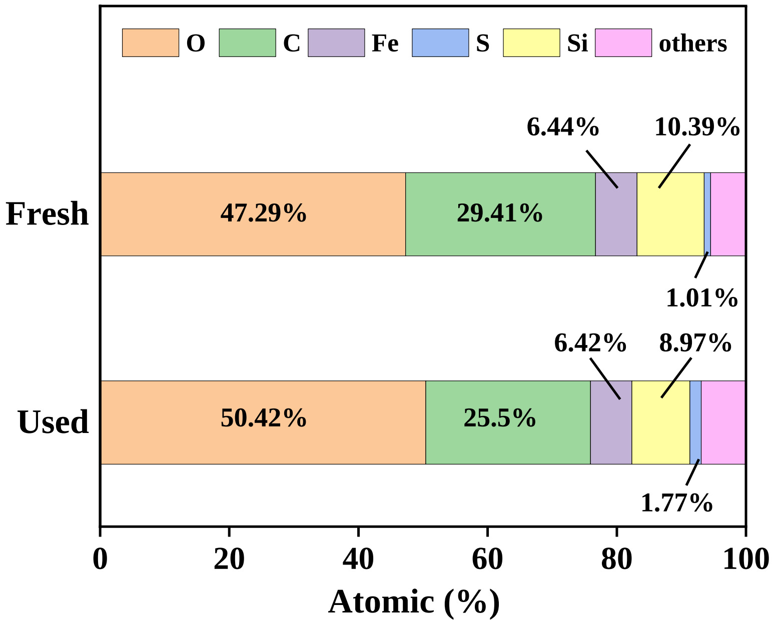

Figure S4. Atom Percentages of B-nZVI/BC before and after reaction in the XPS survey spectra.

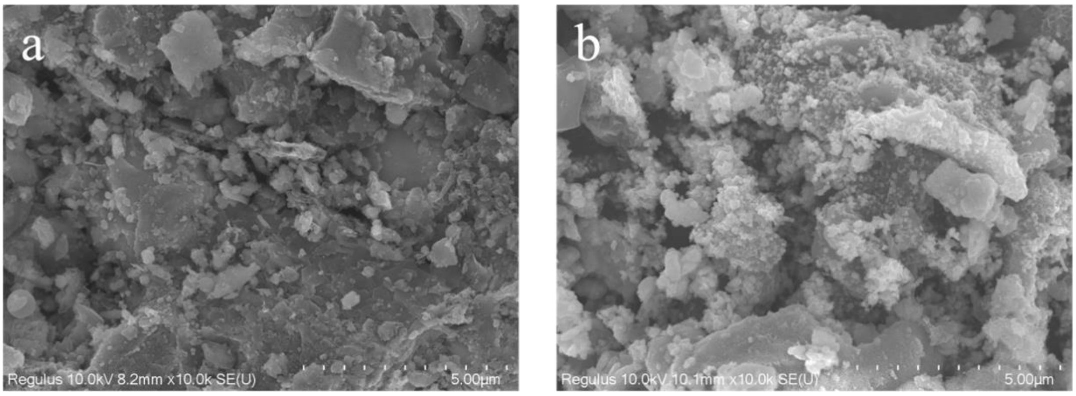

Figure S5. SEM of B-nZVI/BC before (**a**) and after reaction (**b**).

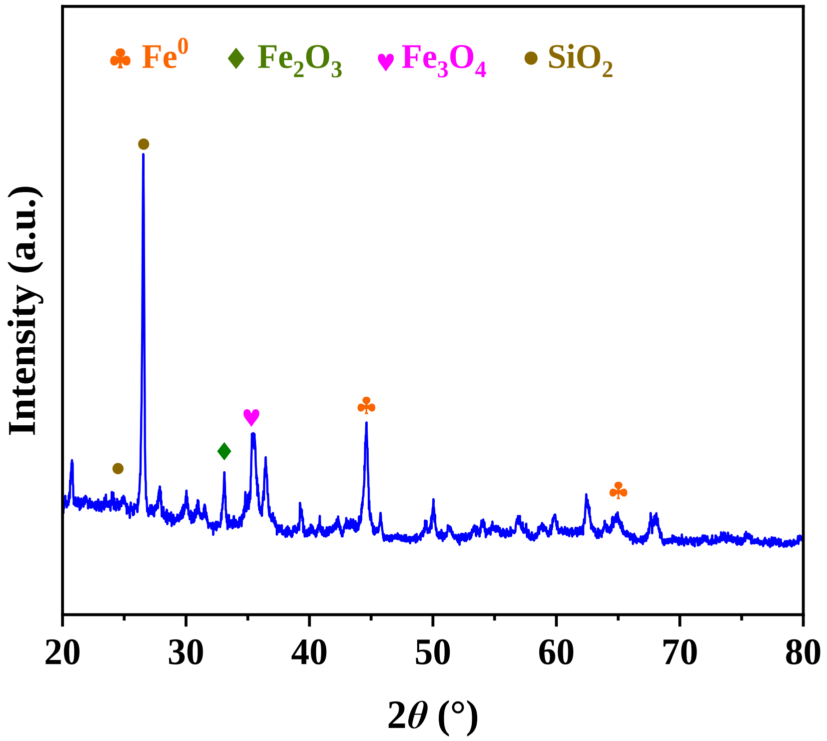

Figure S6. XRD patterns of B-nZVI/BC after reaction.

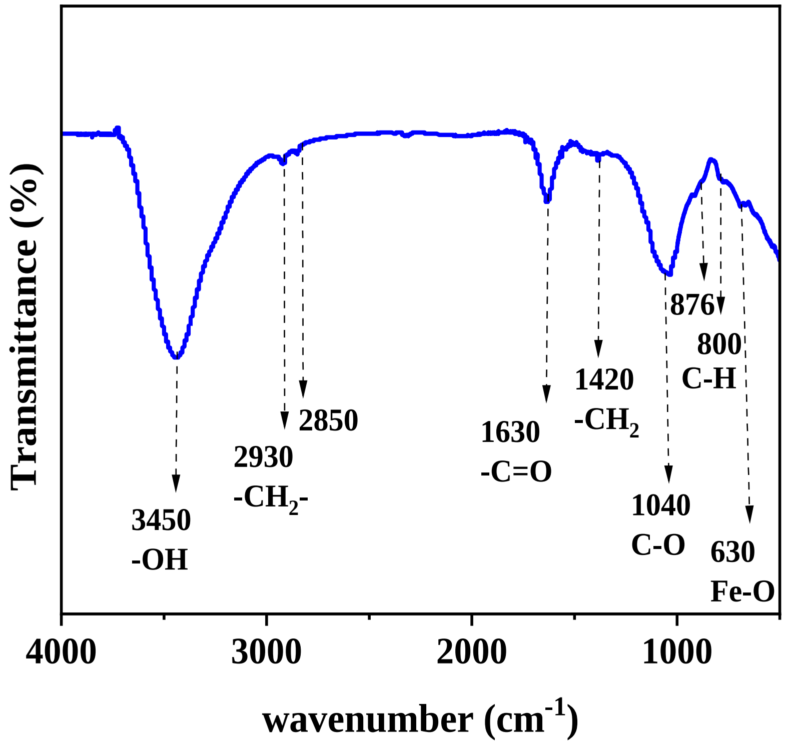

Figure S7. FT-IR of B-nZVI/BC after reaction.

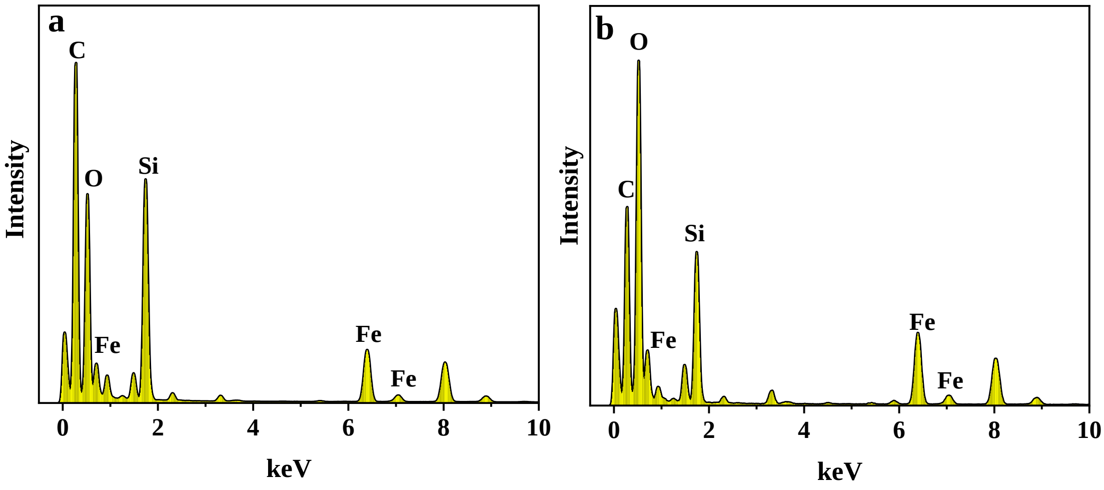

Figure S8. TEM-EDS of B-nZVI/BC before (**a**) and after reaction (**b**).

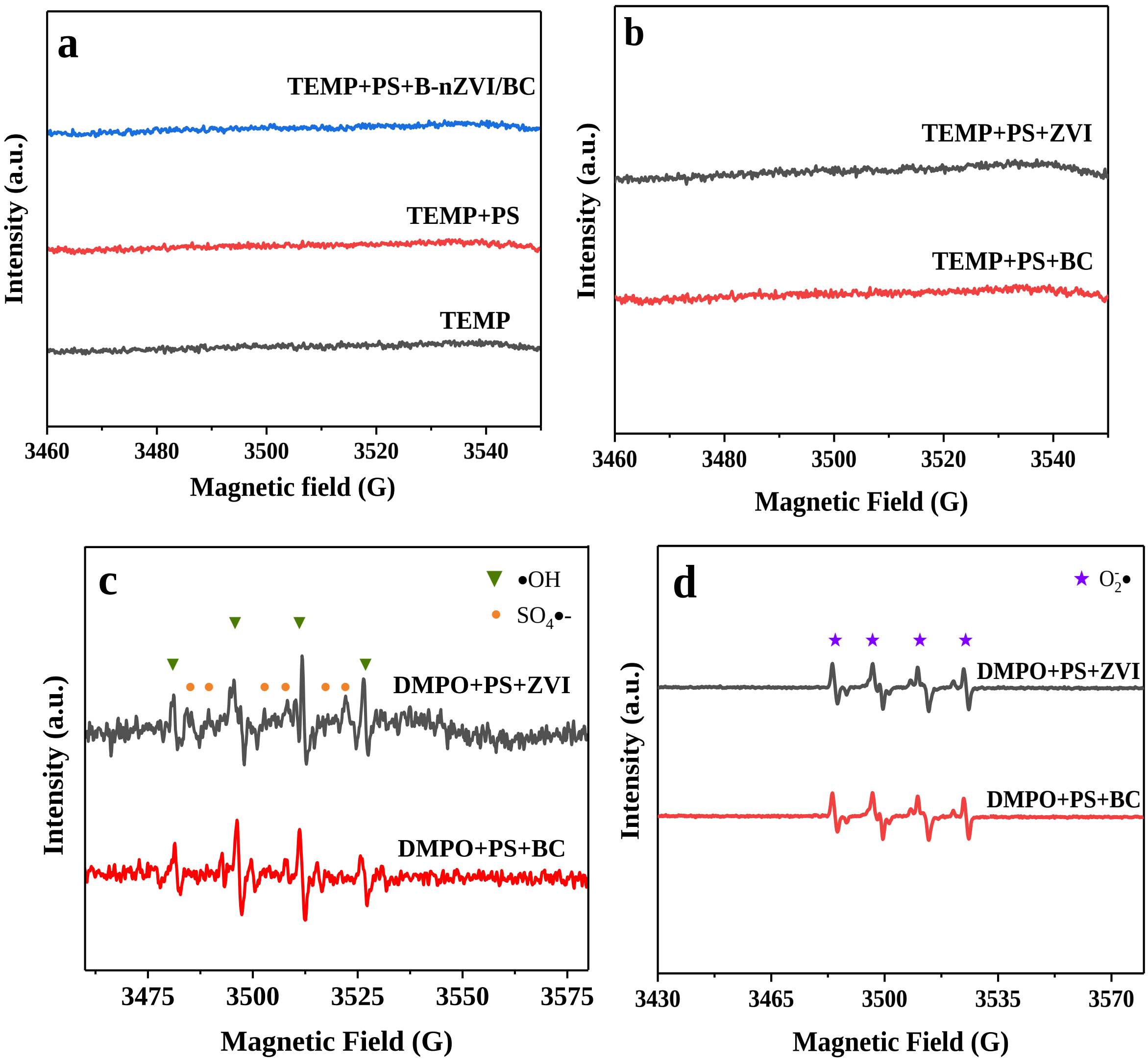

Figure S9. EPR measurements for verify the radicals of $^1O_2$ (**a**), $^1O_2$ (**b**), $SO_4^{\bullet -}$, $\bullet OH$ (**c**) and $O_2^{\bullet -}$ (The EPR spectrometric detection of $O_2^{\bullet -}$ was performed in DMSO solution, which was designed to avoid the influence of $SO_4^{\bullet -}$ and $\bullet OH$) (**d**) in systems of B-nZVI/BC-PS, BC-PS and ZVI-PS. Reaction conditions: $[PS]_0$ = 42.0 mmol L^{-1}, [DMSO]: $[H_2O]$ =9:1 (Volume ratio), [DMPO] = [TEMP] = 200.0 mmol L^{-1}, $[B\text{-}nZVI/BC]_0$ = 4.8 g kg^{-1}, $[ZVI]_0$ = 2.4 g kg^{-1}, $[BC]_0$ = 2.4 g kg^{-1}, pH_0 = 7.49 and T = 25 °C.

a

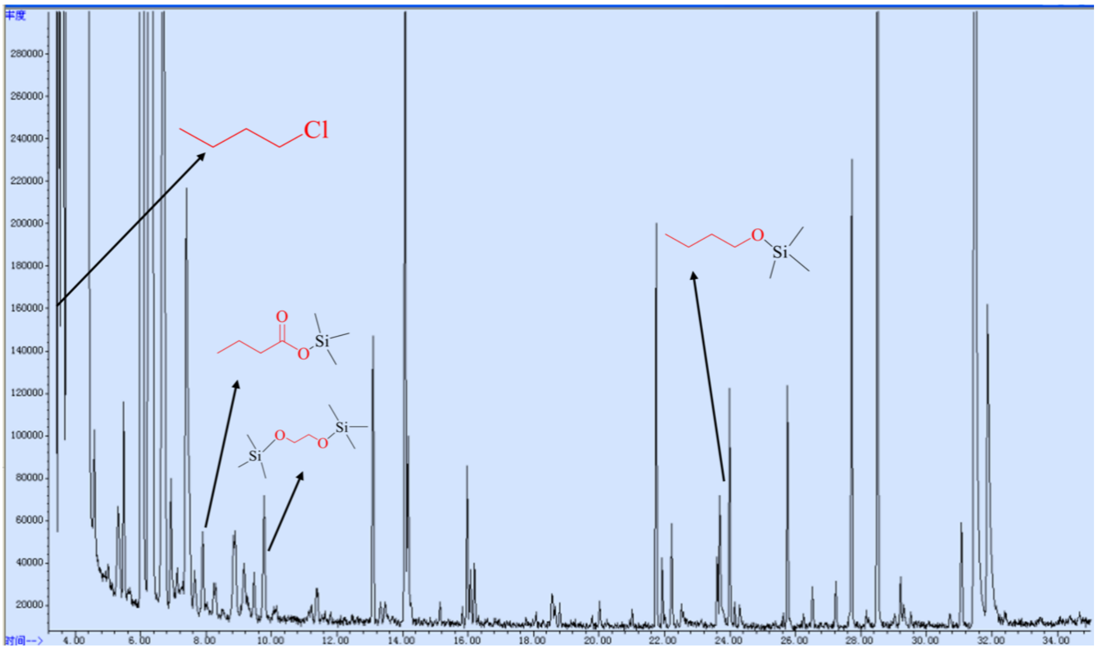
丰度
280000
260000
240000
220000
200000
180000
160000
140000
120000
100000
80000
60000
40000
20000
时间-->
4.00
6.00
8.00
10.00
12.00
14.00
16.00
18.00
20.00
22.00
24.00
26.00
28.00
30.00
32.00
34.00
Cl
O
Si

b

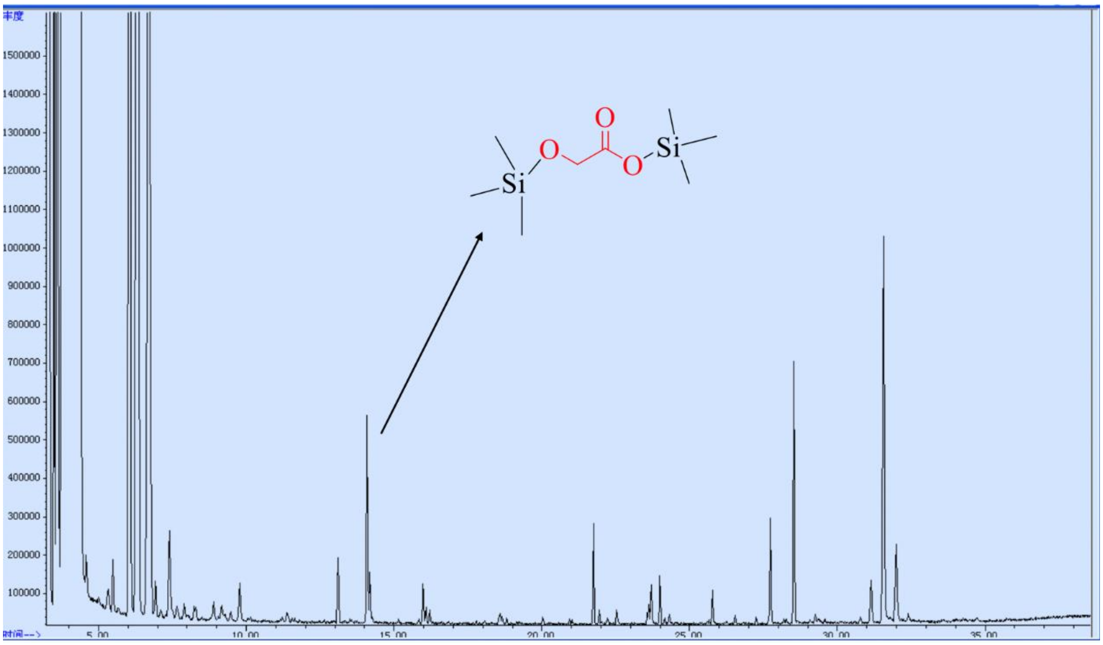
丰度
1500000
1400000
1300000
1200000
1100000
1000000
900000
800000
700000
600000
500000
400000
300000
200000
100000
时间-->
5.00
10.00
15.00
20.00
25.00
30.00
35.00
Si
O
O
O
Si

c

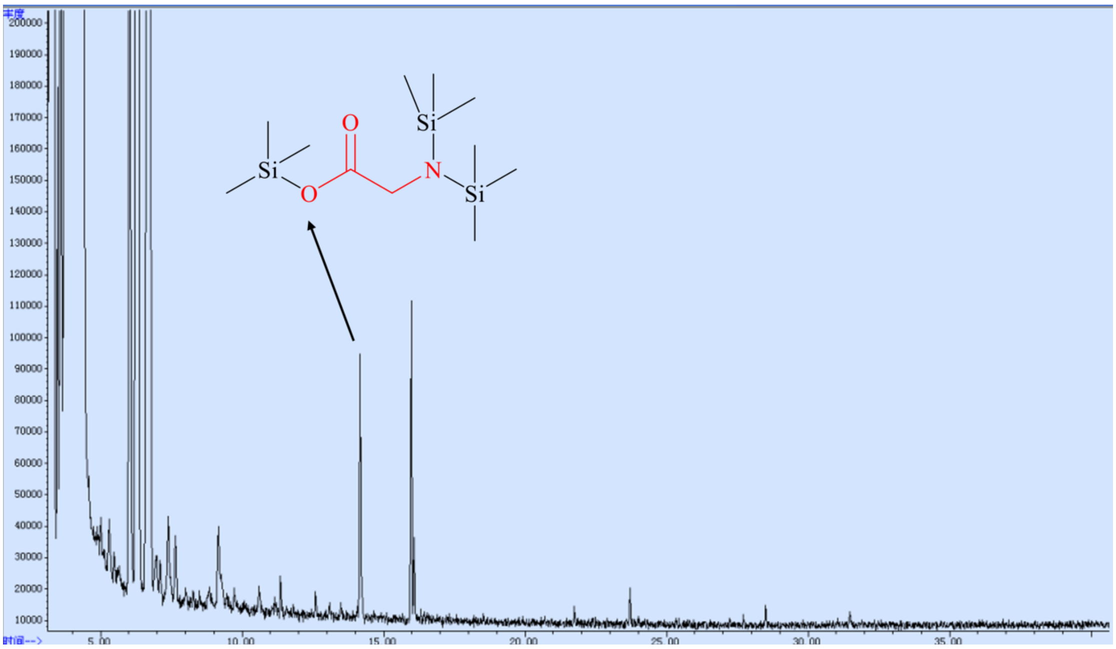
丰度
Si
O
N
Si
Si
O
时间-->
5.00
10.00
15.00
20.00
25.00
30.00
35.00

d

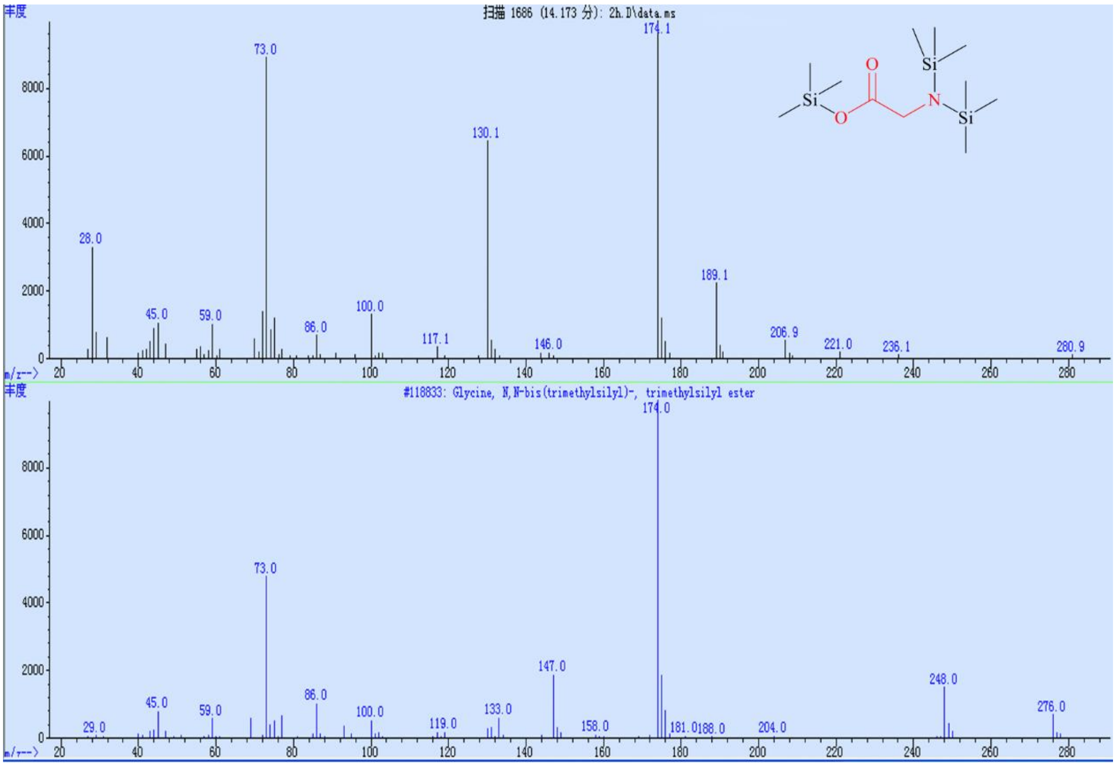
丰度
扫描 1686 (14.173 分): 2h.D\data.ms
174.1
73.0
130.1
28.0
189.1
45.0
59.0
100.0
86.0
117.1
146.0
206.9
221.0
236.1
280.9
m/z-->
丰度
#118833: Glycine, N,N-bis(trimethylsilyl)-, trimethylsilyl ester
174.0
73.0
147.0
248.0
276.0
45.0
86.0
59.0
100.0
133.0
29.0
119.0
158.0
181.0
188.0
204.0
m/z-->

e

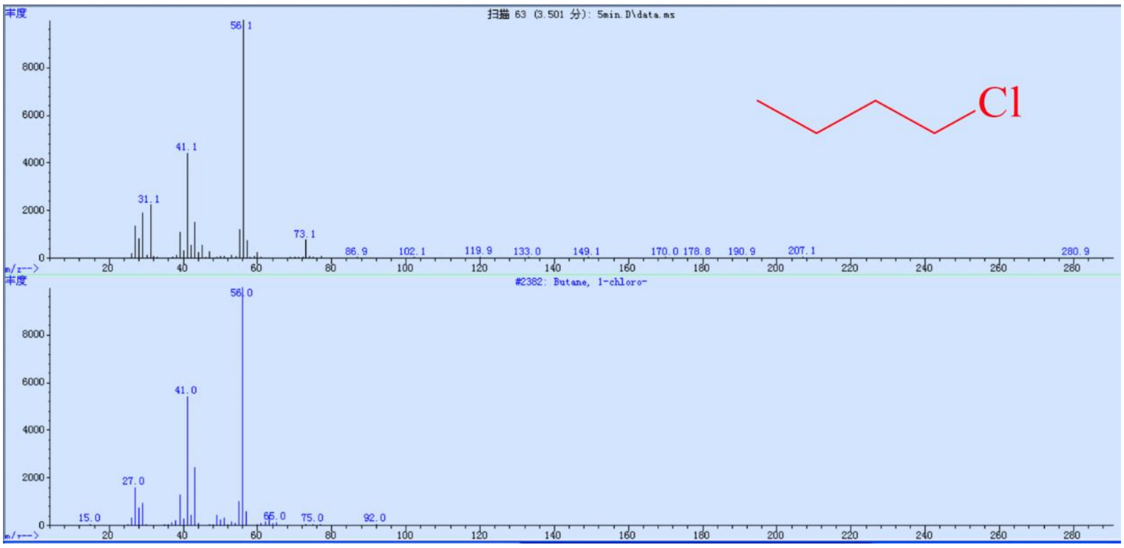
丰度
扫描 63 (3.501 分): 5min.D\data.ms
#2382: Butane, 1-chloro-
Cl

f

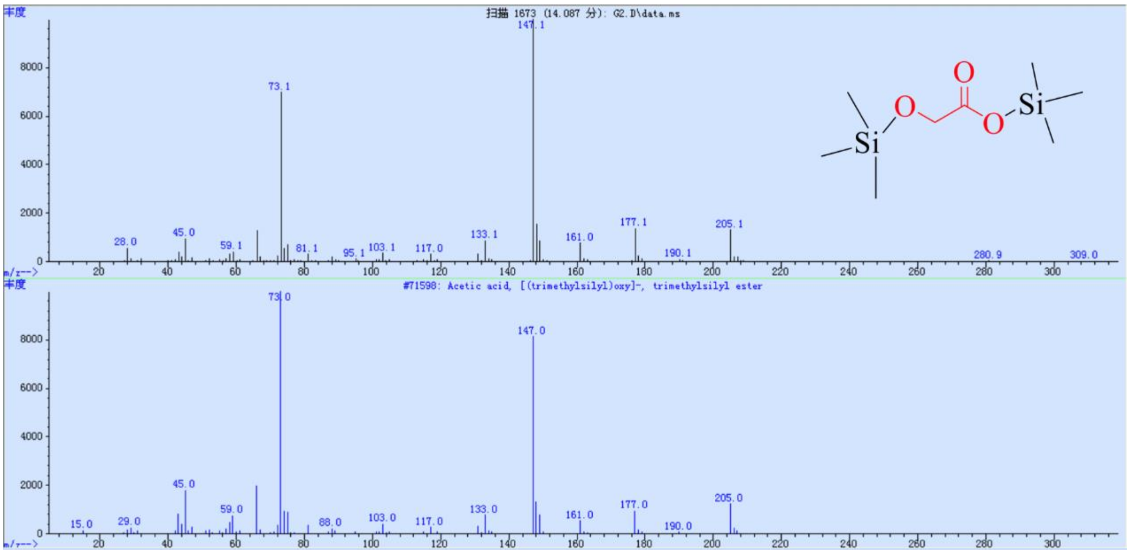
丰度
扫描 1673 (14.087 分): G2.D\data.ms
#71598: Acetic acid, [(trimethylsilyl)oxy]-, trimethylsilyl ester
O
Si

g

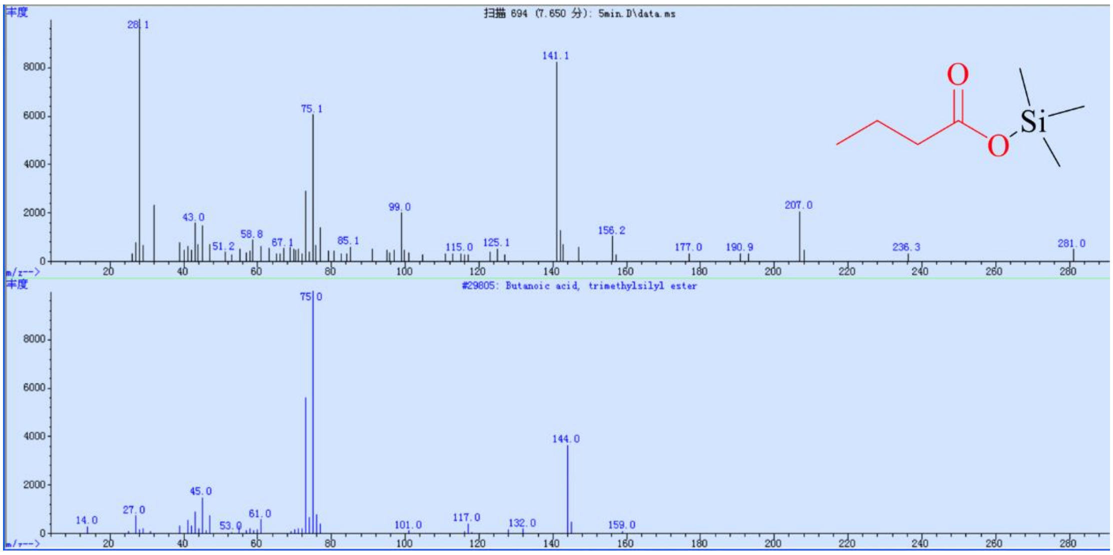
丰度
扫描 694 (7.650 分): 5min.D\data.ms
#29805: Butanoic acid, trimethylsilyl ester
O
Si

h

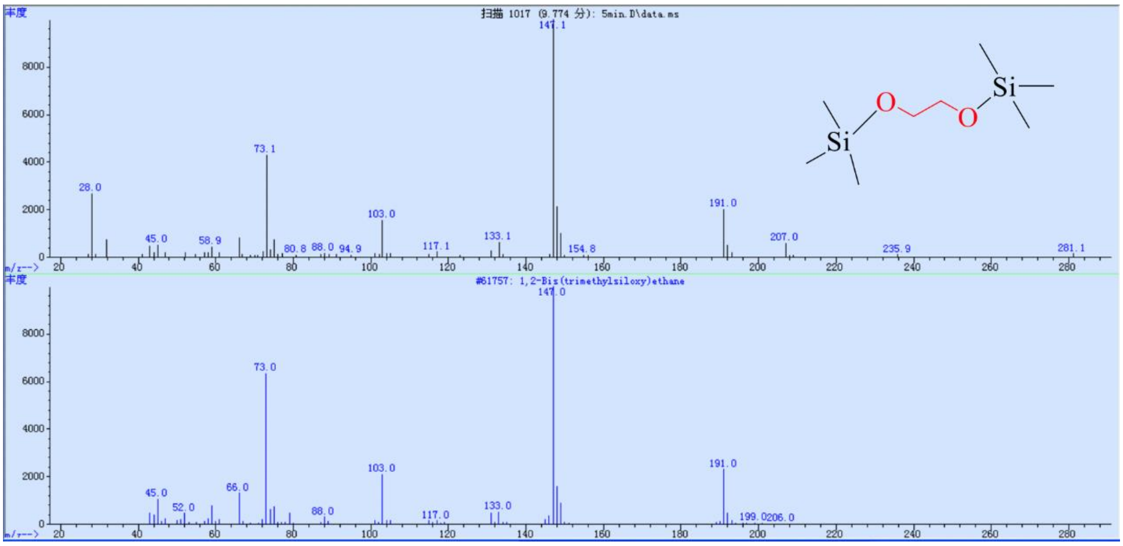

i

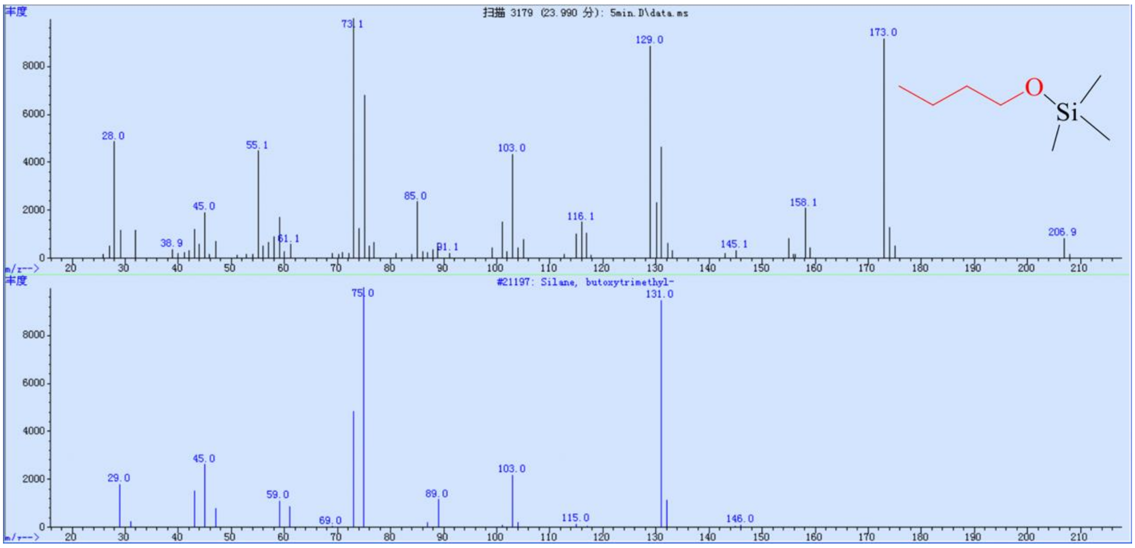

Figure S10. GC-MS ion spectra at the reaction time of 5 min (**a**), 30 min (**b**), 120 min (**c**) and its derivatives from BSTFA-silylated degradation intermediates (**d–i**) of PCA in the B-nZVI/BC-PS system.

References

1. EPA, 2016. EPA/600/R-16/114. Analytical protocol for measurement of extractable semivolatile organic compounds using gas chromatography/mass spectrometry. https://nepis.epa.gov/Exe/ZyPURL.cgi?Dockey=P1011HNL.txt.
2. EPA, 2018. Method 8270E (SW-846). Semivolatile organic compounds by gas chromatography/mass spectrometry (GC/MS). https://www.epa.gov/hw-sw846/sw-846-test-method-8270e-semivolatile-organic-compounds-gas-chromatographymass.
3. Tongarun R.; Luepromchai E.; Vangnai A S. Natural attenuation, biostimulation, and bioaugmentation in 4-chloroaniline-contaminated soil. *Curr Microbiol*. **2008**, *56*, 182-188. 10.1007/s00284-007-9055-y.
